# Structure, optical properties and antimicrobial activities of MgO–Bi_2−*x*_Cr_*x*_O_3_ nanocomposites prepared via solvent-deficient method

**DOI:** 10.1038/s41598-022-14811-9

**Published:** 2022-06-23

**Authors:** Annas Al-Sharabi, Kholod S. S. Sada’a, Ahmed AL-Osta, R. Abd-Shukor

**Affiliations:** 1 Physics Department, Faculty of Applied Science, Thamar University, 87246 Dhamar, Yemen; 2 Department of Applied Physics, Universiti Kebangsaan Malaysia, 43600 Bangi, Selangor Malaysia

**Keywords:** Materials science, Condensed-matter physics

## Abstract

MgO–Bi_2−*x*_Cr_*x*_O_3_ nanocomposites for *x* = 0 and 0.07 were fabricated using the solvent-deficient route. X-ray diffraction method, scanning electron microscopy (SEM), energy dispersive X-ray analysis (EDXA) and UV–Vis spectroscopy were employed to study the properties. The samples were also evaluated for the antibacterial activity. The *x* = 0 sample showed a dominant monoclinic crystalline structure of $\alpha\text{-}{\text{Bi}}_{2}{\text{O}}_{3}$ phase. No peaks attributed to MgO were observed. Cr-doped $\text{MgO}{-}{\text{Bi}}_{2}{\text{O}}_{3}$ in which Bi was substituted showed that ${\text{the tetragonal BiCrO}}_{3}$ phase was also present in the $\text{MgO}{-}{\text{Bi}}_{2}{\text{O}}_{3}$ composite. The Scherrer formula was employed to determine the crystallite size of the samples. The Cr-doped sample showed a decrease in the crystallite size. The microstructures of the non-doped MgO–Bi_2_O_3_ and MgO–Bi_1.93_Cr_0.07_O_3_ composites consisted of micrometer sized grains and were uniformly distributed. Direct transition energy gap, ${E}_{\text{g}}$ decreased from 3.14 to 2.77 eV with Cr-doping as determined from UV–Vis spectroscopy. The Cr-doped $\text{MgO}{-}{\text{Bi}}_{2}{\text{O}}_{3}$ nanocomposites exhibited two energy gaps at 2.36 and 2.76 eV. The antibacterial activity was determined against gram-negative bacteria (*Salmonella typhimurium and Pseudomonas aeruginosa*) and gram-positive bacteria (*Staphylococcus aureus*) by disc diffusion method. Cr-doping led to a decrease in inhibitory activity of MgO–Bi_2−*x*_Cr_*x*_O_3_ nanocomposite against the various types of bacteria.

## Introduction

The unique physical and chemical characteristic of nanoparticles have led to intense research in this field in the last few years^1^. Nanoparticles also have some antibacterial properties due to the inherent dimension, structural and surface characteristics^2^. Nanotechnology can be capitalized to improve the activity of inorganic antibacterial materials. Bismuth (III) oxide or bismuth trioxide with the chemical formula Bi_2_O_3_ is a yellow chemical compound^3^. It is a highly insoluble and thermally stable compound^4^. Bi_2_O_3_ exists in six distinct polymorphs namely *α, β, γ, δ, ε* and *ώ* for monoclinic, tetragonal, body-centered cubic, cubic face centered, orthorhombic, and triclinic forms, respectively. The α-phase is generally formed at a lower temperature (around 873 K) compared to other phases.

Several techniques have been employed to improve the formation of α and β-Bi_2_O_3_ and the thermal stability^5^. Bi_2_O_3_ is a p-type semiconductor with a narrow direct band gap (2.85 eV)^3,6–8^. The ionic radius of Bi^3+^ is 0.103 nm. Nano-Bi_2_O_3_ has good photoluminescence properties, large ionic conductivity and dielectric permittivity, remarkable photoconductivity, non-toxic and excellent catalytic activity^4,9–14^. It is useful in fuel cells, optical coating, optoelectronics, high temperature superconductors and piezoelectric material^9,15–22^. Several Bi_2_O_3_-containing semiconductor compounds have been used in photocatalysis. However, it has a poor photo-quantum efficiency, limited light response range and inferior catalytic ability in the visible spectrum. These problems limited the application of Bi_2_O_3_^2,8,21^.

Bi_2_O_3_–MgO have been fabricated by solvent-thermal method and the photocatalytic activity has been reported. Bi_2_O_3_–MgO with Bi to Mg molar ratio of 2:1 has the narrowest band gap and it was found more active for photocatalytic decolorization of RhB than Bi_2_O_3_ and MgO^23^. CeO_2_–Bi_2_O_3_ nanocomposite was prepared via a two-stage process and the photocatalytic activity has been reported. The results showed that the microstructure and morphology of CeO_2_–Bi_2_O_3_ composite were similar in spite of different inverse proportion. Improved photocatalytic activity was observed in the case of CeO_2_–Bi_2_O_3_ composite catalyst compared to the catalytic activity of pure Bi_2_O_3_ or CeO_2_ powder. The suppression of charge recombination in the composite CeO_2_–Bi_2_O_3_ catalyst led to higher catalytic activity for the degradation of RhB^24^. The ZnO–Bi_2_O_3_ with tunable optical properties and the antibacterial activity have been studied. The formation of the BZO nanocomposites was confirmed by the coexistence of both ZnO and Bi_2_O_3_ phases in diffraction patterns. The decoration of Bi_2_O_3_ nanoparticles on the surface of ZnO nano-cones significantly improved the optical quality. The Bi_2_O_3_ nanoparticles decoration on ZnO nano-cones reduce surfaced defects and increased electron–hole recombination rate which strongly influence the antibacterial activity of BZO nanocomposites^25^. Wu et al. synthesized ZnO–Bi_2_O_3_ nanocomposite by sonochemical route at low-temperature^26^. It is useful to prepare new effective photocatalysts in the visible region. Bi_2_O_3_ has very attractive antimicrobial activity. Generally, Bi_2_O_3_ has low cytotoxicity and present notable antibacterial activity^27–29^.

Magnesium oxide is a white solid mineral. Mg has oxidation state + 2 and its ionic radius is 0.72 Å^3^. Magnesium oxide is a semiconductor/insulator which usually display a cubic structure^30^. The band gap of MgO is around 7.8 eV and this quite large which limited its application. The nano sized magnesium oxide has a lower band gap of 5 eV^31^. Magnesium oxide nanoparticles have high surface reactivity and good chemical and thermal stability^32^. Nano sized MgO has a wide-range of bactericidal property towards gram-positive and gram-negative bacteria^33^. It demonstrates higher mammalian bioactivity and lower toxicity than most metal oxides. Hence, it can be a potential ingredient in drug formulation^34^. Many reports have been published on the synthesis of MgO nanoparticles and nanocomposite due to the wide range of applications^35^. The antibacterial activity lies in the creation of superoxide radicals through the reaction of oxygen with the bacterial surface. The extra electrons are very reactive and can cause damage to the proteins and phospholipids of the bacterial membrane^36^.

Chromium in the trivalent state has an ionic radius of 0.62 Å^37^. Among the various transition metals, chromium is known to improve the structure and optical characteristics of nanocomposites. The antimicrobial activities of chromium (III) exhibits high biological factor because it contains amino acids as ligand.

In this work, MgO–Bi_2−*x*_Cr_*x*_O_3_ (*x* = 0 and 0.07) nanocomposite powders were prepared using a low cost solvent-deficient technique. The X-ray diffraction (XRD) method, scanning electron microscope (SEM) and UV–Vis spectrophotometer were used to study the structure, microstructure and optical properties, respectively. The average crystallite size, *D* was calculated using the Scherrer formula. The antibacterial activity was studied against gram-negative bacteria (*Salmonella typhimurium* and *Pseudomonas aeruginosa*) and gram-positive bacteria (*Staphylococcus aureus*) by means of the disc diffusion method at various concentrations of the prepared nanocomposite.

## Experimental procedure

The MgO, Bi_2_O_3_ and MgO–Bi_2−*x*_Cr_*x*_O_3_ for *x* = 0 and 0.07 nanocomposites were prepared using facile solvent-deficient technique using: Bi(NO_3_)_3_·5H_2_O (≥ 98.5%; Fluka), Mg(NO_3_)_2_·6H_2_O, Cr(NO_3_)_3_·9H_2_O (> 99%; Fluka-Garande) and NaHCO_3_ (> 99%; Fluka). All chemicals were used as received without further purification. The reaction is as follows:

1 $${\text{Mg}}\left( {{\text{NO}}_{{3}} } \right)_{{2}} \cdot {\text{6H}}_{{2}} {\text{O }} + {\text{ 2NaHCO}}_{{3}} \to {\text{ MgO }} + {\text{ 2NO}}_{{2}} + {\text{ 7H}}_{{2}} {\text{O }} + {\text{ Na}}_{{2}} {\text{O}} + {\text{ 2CO}}_{{2}} + {1}/{\text{2O}}_{{2}} ,$$

2 $${\text{2Bi}}\left( {{\text{NO}}_{{3}} } \right)_{{3}} \cdot {\text{5H}}_{{2}} {\text{O }} + {\text{ 6NaHCO}}_{{3}} \to {\text{ Bi}}_{{2}} {\text{O}}_{{3}} + {\text{ 6NO}}_{{2}} + {\text{ 13H}}_{{2}} {\text{O }} + {\text{ 3Na}}_{{2}} {\text{O}} + {\text{ 6CO}}_{{2}} + {3}/{\text{2O}}_{{2}} ,$$

3 $$\left( {{2} - x} \right){\text{Bi}}\left( {{\text{NO}}_{{3}} } \right)_{{3}} \cdot {\text{5H}}_{{2}} {\text{O }} + {\text{ Mg}}\left( {{\text{NO}}_{{3}} } \right)_{{2}} \cdot{\text{6H}}_{{2}} {\text{O }} + x{\text{Cr}}\left( {{\text{NO}}_{{3}} } \right)_{{3}} \cdot {\text{9H}}_{{2}} {\text{O}} + {\text{ 8NaHCO}}_{{3}} \to {\text{ MgO}} - {\text{Bi}}_{{{2} - x}} {\text{Cr}}_{x} {\text{O}}_{{3}} + {\text{ 8NO}}_{{2}} + \, \left( {{2}0 + {4}x} \right){\text{H}}_{{2}} {\text{O }} + {\text{ 4Na}}_{{2}} {\text{O}} + {\text{ 8CO}}_{{2}} + {\text{ 2O}}_{{2}} .$$

Stoichiometrically calculated amounts of metal nitrates were mixed with NaHCO_3_ and grounded together for 20 min in mortar at room temperature. A noticeable degassing reaction due to CO_2_ release was initially observed. The slurry became more viscous with continuous mixing. After drying overnight at room temperature in the mortar, a dark powder was obtained. The powder rinsed using distilled H_2_O and filter flask to wash the powder 3 times. The powders were heated in a box oven for 2 h at 773 K and the temperature was increased or decreased at 10 K/min.

An XD-2 X-ray diffractometer (China) located at the Yemeni Geological Survey and Minerals Resources Board was used to identify the phase. The CuK_α_ radiation was used and the angle 2*θ* was varied from 20° to 70°. Scanning electron micrographs (SEM) and energy dispersive X-ray analyzer from JEOL-JSM 6360 LV (Japan) were used to determine the microstructure and elemental composition. The optical transmission and absorption were investigated using a UV–Vis spectrophotometer (SPECORD 200) at room temperature in the wavelength range of 200–900 nm in the Department of chemistry, college of sciences, Sana’a University.

The antibacterial activity of MgO–Bi_2−*x*_Cr_*x*_O_3_ nanocomposites was assessed against gram-negative bacteria (*Salmonella typhimurium* and *Pseudomonas aeruginosa*) and gram-positive bacteria (*Staphylococcus aureus*) by the disc diffusion method. Biochemical test was used to identity the isolates. The samples were suspended in sterile distilled water and diluted in one-fold serial dilution from the stock solution of 50 mg/ml. Four working dilutions were used for disks impregnation. A sterile filter paper disk with 6 mm diameter was impregnated with 20 μl (10 μl to each side) producing 500, 250, 125, 62.5 μg/disk (S1, S2, S3, S4, respectively). Inoculation by swabs resulted in a homogeneous bacterial lawn on the agar surface. The disks were placed on the surface of the inoculated agar with sterile forceps and incubated at 310 K for 18–20 h. After incubation, the zones of inhibition (ZOI) were determined to the closest mm. For the negative control, distilled water was employed.

## Results and discussion

### XRD analysis

**Figure 1:**
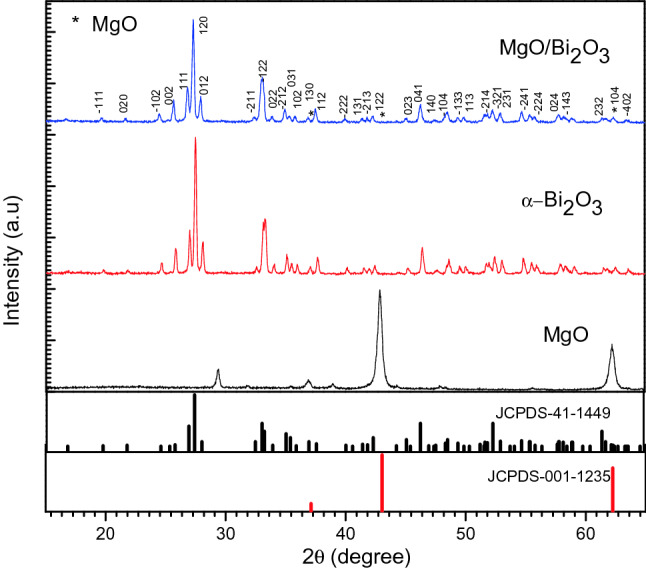
XRD diffraction patterns of $\text{MgO}$, $\alpha{\text{-}}{\text{Bi}}_{2}{\text{O}}_{3}$ and $\text{MgO}{-}\alpha{-}{\text{Bi}}_{2}{\text{O}}_{3}$ nanocomposites.

The crystal structure of $\text{MgO}{-}{\text{Bi}}_{2}{\text{O}}_{3}$ and chromium doped $\text{MgO}{-}{\text{Bi}}_{2}{\text{O}}_{3}$ were confirmed by X-ray diffraction (XRD) analysis. The XRD patterns of the as-prepared $\text{MgO}$, $\alpha{\text{-}}{\text{Bi}}_{2}{\text{O}}_{3}$ and $\text{MgO}{-}{\text{Bi}}_{2}{\text{O}}_{3}$ nanocomposites are exhibited in Fig. 1. The XRD pattern of MgO was dominated by the diffractions typical of the cubic structure of the oxide (space group, Fm-3 m, periclase structure). The diffractions were indexed to the standard JCPDS card number 00–001-1235. An unknown peak was observed near 2*θ* = 30°. The diffractions pattern of $\alpha{\text{-}}{\text{Bi}}_{2}{\text{O}}_{3}$ demonstrated a single phase monoclinic crystalline structure (space group, P21/c), which can be indexed to JCPDS card number 00-041-1449.

The diffraction peaks of the as-prepared $\text{MgO}{-}{\text{Bi}}_{2}{\text{O}}_{3}$ nanocomposites are very similar to those of pure $\alpha{\text{-}}{\text{Bi}}_{2}{\text{O}}_{3}$, but they markedly widen against those of pure $\alpha{\text{-}}{\text{Bi}}_{2}{\text{O}}_{3}$. This implies that the final product possibly has smaller particle size. The XRD pattern of $\text{MgO}{-}{\text{Bi}}_{2}{\text{O}}_{3}$ nanocomposites verified the existence of ${\alpha}{\text{-}}{\text{Bi}}_{2}{\text{O}}_{3}$ monoclinic phase in the single matrix. The peaks can be well matched with JCPDS card number 00-041-1449 for monoclinic crystalline structure $\alpha{\text{-}}{\text{Bi}}_{2}{\text{O}}_{3}$ (space group, P21/c). The (120) peak was the most dominant. However, there was no peaks attributed to MgO probably due to the small amount. However, its presence has been confirmed by EDX spectrum. Bi^3+^ (coordination number = 6) has ionic radius 1.03 Å while Mg^2+^ (coordination number = 6) is only 0.72 Å. The presence of MgO may have suppressed the growth of ${\text{Bi}}_{2}{\text{O}}_{3}$ crystal. The incorporation of Mg^2+^ in the lattice of ${\text{Bi}}_{2}{\text{O}}_{3}$ resulted in a reduction of the lattice parameters of the monoclinic phase. The presence of ${\text{Bi}}_{2}{\text{O}}_{3}$ also suppressed the crystallization of MgO^23^. Diffraction peaks other than ${\text{Bi}}_{2}{\text{O}}_{3}$ werenot observed. This indicated that there was no impurity in the $\text{MgO}{-}{\text{Bi}}_{2}{\text{O}}_{3}$ nanocomposites.

**Figure 2:**
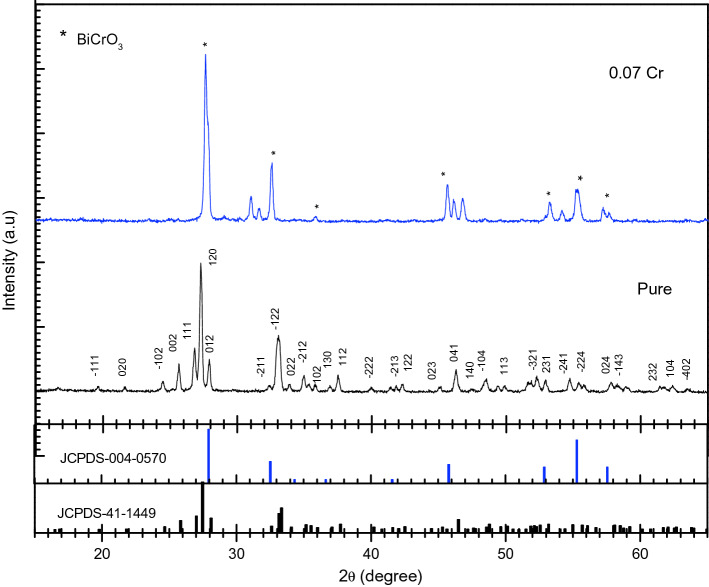
XRD diffraction patterns of MgO–Bi_2−*x*_Cr_*x*_O_3_ nanocomposites for *x* = 0 and 0.07.

The XRD patterns of $\text{MgO}{-}{\text{Bi}}_{2}{\text{O}}_{3}$ and Cr-doped $\text{MgO}{-}{\text{Bi}}_{2}{\text{O}}_{3}$ nanocomposite for 2*θ* = 15°–65° are presented in Fig. 2. Doping with Cr (*x* = 0.07) exhibited new peaks at 27.9°, 32.6°, 41.6°, 52.9°, 55.3° and 57.5° (marked as * in Fig. 2) which correspond to (211), (220), (302), (420), (332) and (422) planes, respectively of the tetragonal ${\text{BiCrO}}_{3}$ phase (JCPDS card number 00-004-0570)^38^. Other Cr related peaks were observed which indicated that the Cr may also reside as interstitial ions or resided at the vacancies. This suggests a new ${\text{BiCrO}}_{3}$ phase was present in the $\text{MgO}{-}{\text{Bi}}_{2}{\text{O}}_{3}$ composite. Moreover, Cr ions may be separated from $\text{MgO}{-}{\text{Bi}}_{2}{\text{O}}_{3}$ and resulted in a new phase with the Bi and O ions.

**Figure 3:**
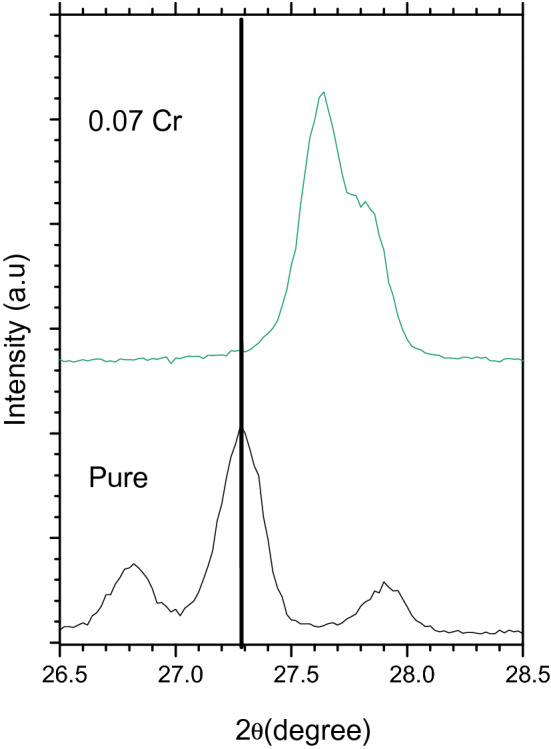
XRD patterns showing a slight peak shift toward higher angles 2*θ* of MgO–Bi_2−*x*_Cr_*x*_O_3_ nanocomposites for *x* = 0 and 0.07.

Cr-doped $\text{MgO}{-}{\text{Bi}}_{2}{\text{O}}_{3}$ showed a wider line broadening compared to the pure ${\text{MgO}}{-}{\text{Bi}}_{2}{\text{O}}_{3}$ indicating that Cr ions may have resided in the $\text{MgO}{-}{\text{Bi}}_{2}{\text{O}}_{3}$ lattice^39^. The shifting of $\text{MgO}{-}{\text{Bi}}_{2}{\text{O}}_{3}$ peaks with Cr-doping can be observed in Fig. 3. The higher angle shift suggested the shrinkage of the *c*-axis. The intensity was reduced for $\text{MgO}{-}{\text{Bi}}_{2}{\text{O}}_{3}$ added Cr^3+^ which has a smaller ionic radius than Bi^3+^^39^. The intensity for the *x* = 0.07 sample was higher which showed that the crystal grew in one crystallographic direction with the lowest surface energy. Hence, by doping the crystallinity and crystal orientation along the *c*-axis can be controlled.

The unit cell parameters *a, b, c* and *β* for monoclinic α˗Bi_2_O_3_ structure were calculated using the following relation:

4 $$\frac{1}{{d}^{2}}= \frac{1}{{\text{sin}}^{2}\beta }\left(\frac{{h}^{2}}{{a}^{2}}+\frac{{k}^{2}{\text{sin}}^{2}\beta }{{b}^{2}}+\frac{{l}^{2}}{{c}^{2}}-\frac{2hl\text{cos}\beta }{\mathit{ac}}\right).$$

The cubic MgO lattice parameters were calculated using:

5 $$\frac{1}{{d}^{2}}= \left(\frac{{h}^{2}+{k}^{2}+{l}^{2}}{{a}^{2}}\right),$$

where *d* is the spacing between planes and *h, k,* and *l* are the Miller indices. The volume (*V*) for the monoclinic phase was calculated using *V* = *abc* × sin *β* and for the cubic structure *V* = *a*^3^ (Table 1). The lattice constants and volume of tetragonal ${\text{BiCrO}}_{3}$ were calculated using^40^:

6 $$\frac{1}{{d}^{2}}= \left(\frac{{h}^{2}+{k}^{2}}{{a}^{2}}+\frac{{l}^{2}}{{c}^{2}}\right),$$

7 $$V = a^{2} c.$$

**Table 1:** Structural parameters of MgO–Bi_2−*x*_Cr_*x*_O_3_ nanocomposite for *x* = 0 and 0.07.

	Oxides	*a*(Å)	*b*(Å)	*c*(Å)	*β*(°)	Volume (Å)	Density (× 10^3^ kg/m^3^)	*d*-spacing (Å)
JCPDS 011–345	MgO	4.203	–	–	–	74.25	3.56	2.420
JCPDS014-1449	Bi_2_O_3_	5.849	8.169	7.512	112.94	330.5	9.36	3.255
JCPDS004-0570	BiCrO_3_	7.770	7.770	8.080	–	487.8	8.41	3.190
MgO (Pure)	MgO (111)	4.215	–	–	–	748.8	3.58	2.433
Bi_2_O_3_ (Pure)	Bi_2_O_3_ (120)	5.859	8.184	7.537	113.12	332.4	9.31	3.244
*x* = 0	Bi_2_O_3_ (120)	5.872	8.227	7.363	113.52	326.1	9.49	3.266
*x* = 0.07	Bi_2_O_3_ (120)	–	–	–	–	–	–	–
	BiCrO_3_(211)	7.762	7.762	8.364	–	503.9	8.15	3.226

The mass density, ρ for the cubic, monoclinic and tetragonal phase were calculated using $\rho =(Z\times M)/(N\times {a}^{3}) \text{g }{\text{cm}}^{-3}$ where *Z* is the atom number per unit cell, *M* is the molecular mass (g/mol) and *N* is Avogadro’s number^41^. The *d*-spacing was calculated using 2*d*sin*θ* = *nλ*, where *θ* is the angle of reflection, *n* is order of reflection and *λ* is the incident radiation wavelength. The substitution of Cr^3+^ in $\text{MgO}{-}{\text{Bi}}_{2}{\text{O}}_{3}$ resulted in an increase in the unit cell volume and inter-planar distance (Table 1). The Debye Scherrer formula:

8 $$D= 0.9\lambda /\left(\varphi \text{ cos}\theta \right),$$

was employed to determine the average crystallite size from the XRD data of $\text{MgO}$, $\alpha{\text{-}}{\text{Bi}}_{2}{\text{O}}_{3}$ and MgO–Bi_2−*x*_Cr_*x*_O_3_. The micro-strain, ε was calculated using^42^:

9 $$\varepsilon =\varphi /(4 \text{tan}\theta ),$$

where *φ* is the full-width at half-maximum (FWHM), *θ* is where the peak is and *λ* = 1.5406 Å. The dislocation density, *δ* = 1/*D*^2^ due to crystal imperfections was also determined. The crystallite size of undoped $\text{MgO}{-}{\text{Bi}}_{2}{\text{O}}_{3}$ nanocomposites was 36 nm and decreased to 23 nm for Cr-doped $\text{MgO}{-}{\text{Bi}}_{2}{\text{O}}_{3}$ (Table 2). The decrease was possibly due to segregation of Cr on $\text{MgO}{-}{\text{Bi}}_{2}{\text{O}}_{3}$ surface as a result of the difference in the radius of Cr^3+^ and Bi^3+^, in addition to the substitution which restricted the growth of $\text{MgO}{-}{\text{Bi}}_{2}{\text{O}}_{3}$ crystal^43^.

**Table 2:** Average crystallite size, lattice strain and dislocation density determined using Debye Scherrer formula of MgO–Bi_2−*x*_Cr_*x*_O_3_ nanocomposite for *x* = 0 and 0.07.

	Oxides	*D* (nm)	Lattice strain *ε* × 10^–3^	Dislocation density *δ* × 10^–4^ (n m^−2^)
MgO (Pure)	MgO (111)	17	5.56	34.36
Bi_2_O_3_ (Pure)	Bi_2_O_3_ (120)	43	3.41	5.46
*x* = 0	Bi_2_O_3_ (120)	36	4.07	7.69
*x* = 0.07	Bi_2_O_3_ (120)	–	–	–
	BiCrO_3_(211)	23	6.20	18.35

The increase of FWHM in Cr-doped samples indicated the reduction of the crystallite size. The same result for micro strain variations was also observed. A smaller crystallite size gave higher strain which increased with decreased in crystallite size. The decrease in strain for larger crystallite size is a result of the decrease in the surface area of the nanocomposites. In addition, *δ* increased with a decrease in the grain size and vice-versa^44^.

### Microstructure

**Figure 4:**
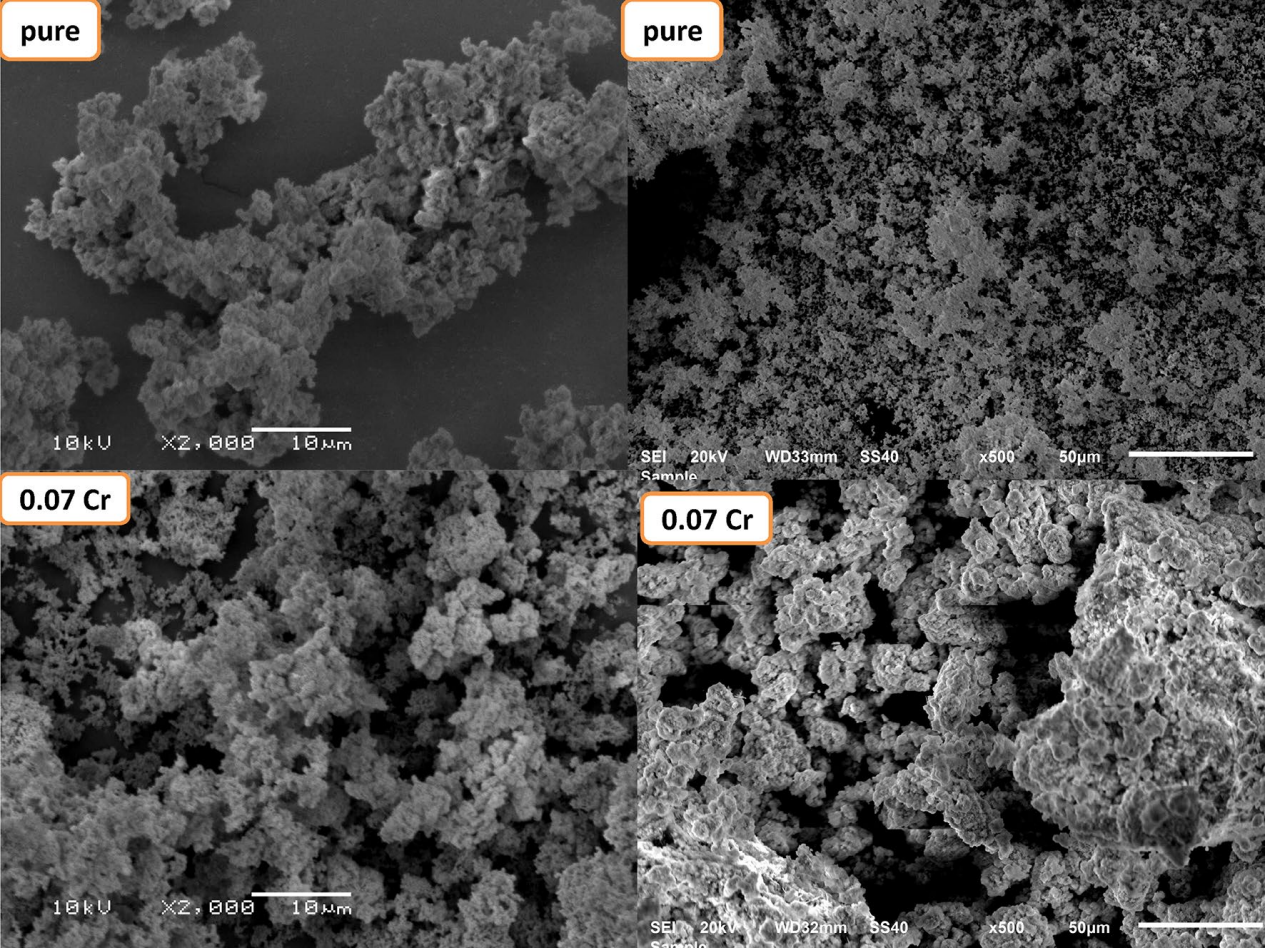
SEM image of MgO–Bi_2−*x*_Cr_*x*_O_3_ nanocomposite for *x* = 0 and 0.07.

Figure 4 shows the micrographs of $\text{MgO}{-}{\text{Bi}}_{2-x}{\text{Cr}}_{x}{\text{O}}_{3}$ for *x* = 0 and 0.07. $\text{MgO}{-}{\text{Bi}}_{1.93}{\text{Cr}}_{0.07}{\text{O}}_{3}$ showed a homogeneous particle distribution with rough surface. The pure and Cr-doped $\text{MgO}{-}{\text{Bi}}_{2}{\text{O}}_{3}$ showed spherical particle distributed uniformly. The Cr-doped sample grain size decreased because Cr^3+^ diffused evenly at the different sites. Hence, pure $\text{MgO}{-}{\text{Bi}}_{2}{\text{O}}_{3}$ showed larger grain size, while the Cr-doped showed smaller size which agreed with the XRD calculations.

The crystallite size of $\text{MgO}{-}{\text{Bi}}_{1.93}{\text{Cr}}_{0.07}{\text{O}}_{3}$ calculated from the XRD data varies from 320 to 700 nm. Particles aggregation is important in determining the morphology and crystalline structure of the samples. The crystallite size determined using the XRD patterns was not the same as the SEM. In SEM the grain size were estimated from the clear grain boundaries. However, the crystalline surface area which diffracts the X-rays effectively were used to determine the crystallite size^45^.

**Figure 5:**
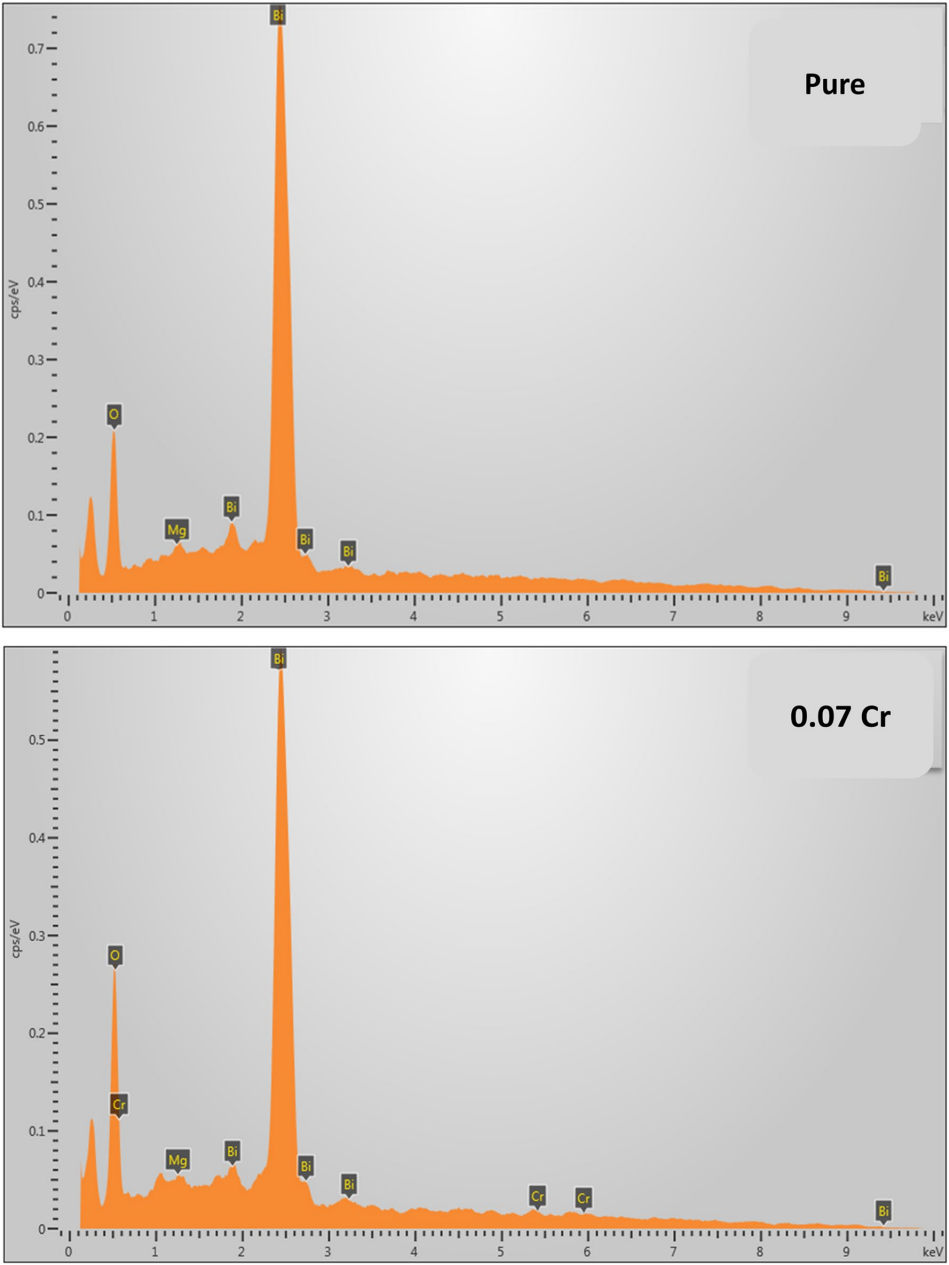
EDXA spectra of MgO–Bi_2−*x*_Cr_*x*_O_3_ nanocomposite for *x* = 0 and 0.07.

From the energy dispersive X-ray analyzer (EDXA), the peaks belonging to Bi, Mg, O and Cr with the expected composition were observed in the spectra (Fig. 5). The EDXA confirmed the elements present in the $\text{MgO}{-}{\text{Bi}}_{1.93}{\text{Cr}}_{0.07}{\text{O}}_{3}$. No other addition impurity peaks was observed which indicated the formation of $\text{MgO}{-}{\text{Bi}}_{1.93}{\text{Cr}}_{0.07}{\text{O}}_{3}$ nanocomposite. In the Cr-doped sample, the Cr content was 0.4%. In the $\text{MgO}{-}{\text{Bi}}_{2}{\text{O}}_{3}$ sample, the elemental compositions of Bi, Mg and O were 88.7%, 0.4% and 10.9%, respectively. In the Cr-doped sample, the Cr content was 0.8%. In the $\text{MgO}{-}{\text{Bi}}_{2}{\text{O}}_{3}$ sample, the elemental compositions of Bi, Mg and O were 83.4%, 3.1% and 13.5%, respectively. In the Cr-doped $\text{MgO}{-}{\text{Bi}}_{2}{\text{O}}_{3}$, the compositions of Bi, Mg and O were 82.2%, 3.2% and 13.8%, respectively. The annul tendency in the chemical composition of Cr-doped samples may be due to smaller ionic radius of Cr^3+^. The slight variation in Cr was also likely a result of dilution of the ions in $\text{MgO}{-}{\text{Bi}}_{2}{\text{O}}_{3}$^46^.

### Optical properties

#### Optical absorption and transmission spectra

**Figure 6:**
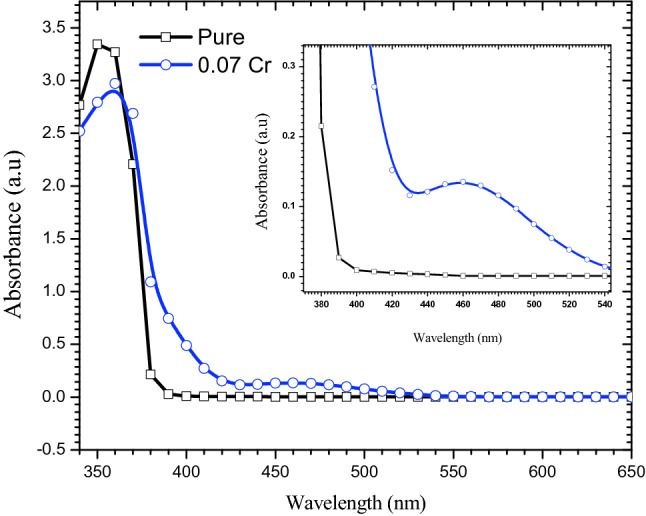
Absorbance versus wavelength of MgO–Bi_2−*x*_Cr_*x*_O_3_ nanocomposite for *x* = 0 and 0.07.

The UV–Visible spectra of $\text{MgO}{-}{\text{Bi}}_{2}{\text{O}}_{3}$ and $\text{MgO}{-}{\text{Bi}}_{2-x}{\text{Cr}}_{x}{\text{O}}_{3}$ (*x* = 0.07) showed a strong absorption in the ultraviolet region (Fig. 6). With chromium doping the band edge showed a slight red shift. This shift was due to sp–d exchange interaction between the band electron and localized *d* electrons of Cr^3+^^47^. The $\text{MgO}{-}{\text{Bi}}_{2-x}{\text{Cr}}_{x}{\text{O}}_{3}$ (*x* = 0.07) showed the main maximum peak at 360 nm, and another peak at 462 nm which was due to the new phase ${\text{BiCrO}}_{3}$. The absorption bands decreased with Cr-doping. The red shift was a result of the smaller particle size^48^.

**Figure 7:**
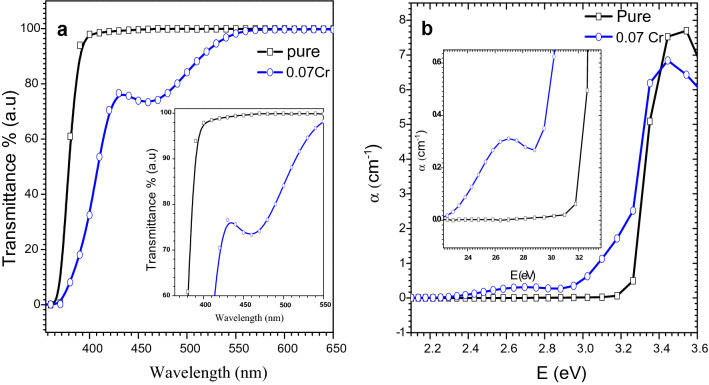
(**a**) Transmittance as a function of wavelength of MgO–Bi_2−*x*_Cr_*x*_O_3_ nanocomposite for *x* = 0 and 0.07 and (**b**) absorption coefficient versus photon energy for *x* = 0 and 0.07.

The transmission spectrum of Cr-doped $\text{MgO}{-}{\text{Bi}}_{2}{\text{O}}_{3}$ with *x* = 0.07 from 350–650 nm is shown in Fig. 7. The optical transparency of $\text{MgO}{-}{\text{Bi}}_{2}{\text{O}}_{3}$ was about 99% in the visible region. The transparency of the nanocomposite decreased Cr-doping due to scattering and absorption from the defects on the surface^49^. The transmittance decreased in the visible range with Cr-doping. This was due to the electrons in the outer orbits which absorbed the energy of the incident light where the electrons was excited to higher levels. No emission of radiation was involved because the excited electron occupied vacant states in the allowed bands. Thus, part of the incident light was absorbed and did not penetrate the material^50^.

The decrease in optical transmittance was due to the grain boundaries because the Cr-doped $\text{MgO}{-}{\text{Bi}}_{2}{\text{O}}_{3}$ exhibited a smaller grain size and higher density of grain boundary. This led to an increase in scattering. These transitions took place in direct and indirect transitions. The moderate transmittance in the long wavelength UV–Vis range is suitable for optoelectronic application including window layers. A low transmittance in the low wavelength region near UV (360 nm) was observed with further increase towards the higher wavelength region. The sudden transmittance drop near the UV region was due to absorption of light through electronic excitation^51^.

#### Absorption coefficient

The absorption coefficient for $\text{MgO}{-}{\text{Bi}}_{2}{\text{O}}_{3}$ and Cr-doped $\text{MgO}{-}{\text{Bi}}_{2}{\text{O}}_{3}$ versus photon energy (*E*) and shown in Fig. 7. The absorption coefficient, *α* can be calculated from the transmittance, *T* and photon absorbance, *A*. The *α* values below and near the edge of each curve was calculated using Beer Lambert’s equation^52^:

10 $$I \, = \, I_{{\text{o}}} {\text{e}}^{ - \alpha t} ,$$

where *I* is instantaneous photon intensity, *I*_o_ is initial intensity and *t* is the thickness of the cuvette. *α* can be calculated using:

11 $$\alpha (\lambda ) = \left( \frac{1}{t} \right) \left(\text{ln} \frac{{I}_{o}}{I} \right) =\left(\frac{1}{t}\right)\left(\text{ln} \frac{1}{T}\right)= \frac{2.303 A}{t}.$$

The absorption coefficient decreased exponentially as the wavelength was increased (Fig. 7b). This could be due to the presence of electric fields within the crystal, strain induced imperfection and lattice deformation, and phonons inelastic scattering of carriers^53^. The absorption coefficient of ${\text{MgO}}{-} {{\text{Bi}}_{2 - {\text{x}}}} {{\text{Cr}}_{\text{x}}} {{\text{O}}_{3}}$ was increased with the Cr-doping. This was due to the increase in the charge carriers resulting in the increase of absorbance and absorption coefficient. The highest *α* was observed in the UV region. *α* decreased in the low energy regions because the probability of excitation from the valence to conduction band was very small. The probability increased at the edge of the absorbance toward the higher energy^42^.

#### Refractive index and extinction coefficient

The refractive index, *n* can be written as^54^:

12 $$n= \frac{1+{R}^{2}}{1-{R}^{2}},$$

where, *R* is the reflection. The extinction coefficient, *k* is the amount of light lost due to absorption and scattering in the material. *α* and the incident photon wavelength can be used to determine *k*^54^:

13 $$k= \frac{{\upalpha \uplambda }}{4\uppi }.$$

**Figure 8:**
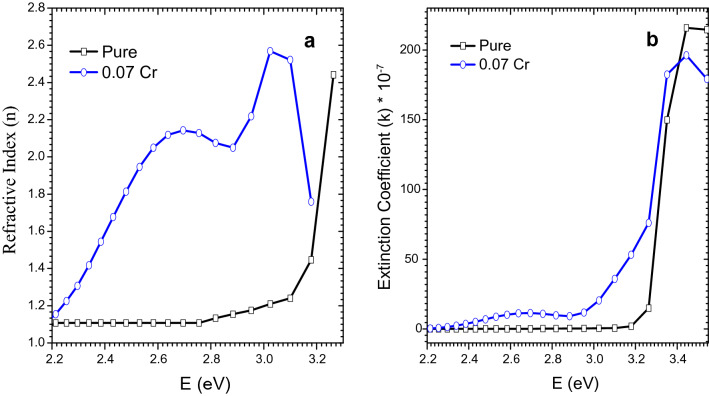
(**a**) Refractive index and (**b**) extinction coefficient versus wavelength of MgO–Bi_2−*x*_Cr_*x*_O_3_ nanocomposite for *x* = 0 and 0.07.

The *n* and *k* versus photon energy plots of ${\text{MgO}}{-} {{\text{Bi}}_{2 - {\text{x}}}} {{\text{Cr}}_{\text{x}}} {{\text{O}}_{3}}$ for *x* = 0 and 0.07 are shown in Fig. 8a,b, respectively. *n* and *k* tend to increase with Cr-doping due to the random grain’s orientation and voids in the sample. Surface roughness can increase the optical scattering which can give rise to an increase in the refractive index^55^.

*n* for Cr-doped sample decreased with a decrease in photon energy from 3.2 to 2.8 eV due to the low absorption coefficient and high transmission^56^ from 388 to 443 nm. This is in good agreement with transmittance and absorption coefficient spectra (Fig. 7a,b). The value of *n* in the visible region was also determined at a specific photon energy such (2.8 eV) for all samples where *n* showed the lowest value. The refractive index showed two distinct regions: an anomalous dispersion at lower energy and normal dispersion at higher energy (Fig. 8a).

The extinction coefficient, *k* of ${\text{MgO}}{-} {{\text{Bi}}_{2 - {\text{x}}}} {{\text{Cr}}_{\text{x}}} {{\text{O}}_{3}}$ nanocomposites was enhanced with increase in photon energy. *k* was very low in the absorption region which indicated the homogeneity of the particles. *k* can be determined from *α* as a function of the energy gap. The *k* value was close to zero which indicated that the nanocomposites were transparent in the visible region. Figure 8b shows that *k* = 0.07 for the Cr-doped $\text{MgO}{-}{\text{Bi}}_{2}{\text{O}}_{3}$ sample and this was higher when compared with the non-doped sample.

#### Optical conductivity

**Figure 9:**
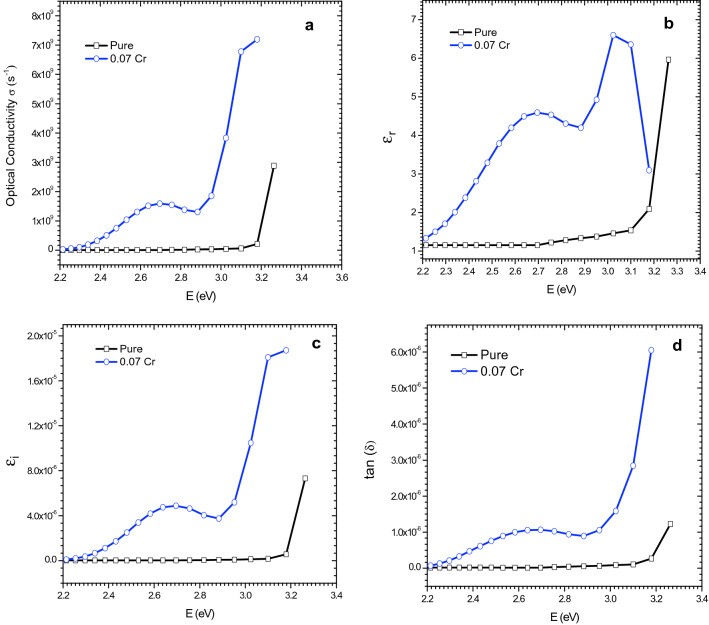
(**a**) Optical conductivity, (**b**) real dielectric constant, (**c**) imaginary dielectric constant and (**d**) loss factor versus photon energy of MgO–Bi_2−*x*_Cr_*x*_O_3_ nanocomposite for *x* = 0.00 and 0.07.

The optical conductivity, ${\sigma }_{opt}$ can be written as^42^:

14 $${\sigma }_{opt}=\frac{\alpha nc}{4\pi },$$

where, *c* is light speed. The optical conductivity versus photon energy for ${\text{MgO}}{-} {{\text{Bi}}_{2 - {\text{x}}}} {{\text{Cr}}_{\text{x}}} {{\text{O}}_{3}}$ (*x* = 0 and 0.07) nanocomposites which indicates the free charges are shown in Fig. 9a^57,58^. Cr-doping enhanced the optical conductivity substantially. It also increased pronouncedly above h*ν* = 2.9 eV for the ${\text{MgO}}{-} {{\text{Bi}}_{2 - {\text{x}}}} {{\text{Cr}}_{\text{x}}} {{\text{O}}_{3}}$ nanocomposites. This was due to the variations of *α* in the ${\text{MgO}}{-} {{\text{Bi}}_{2 - {\text{x}}}} {{\text{Cr}}_{\text{x}}} {{\text{O}}_{3}}$ nanocomposites. The larger optical conductivity appeared in the visible range was due to the energy gap of the ${\text{MgO}}{-} {{\text{Bi}}_{2 - {\text{x}}}} {{\text{Cr}}_{\text{x}}} {{\text{O}}_{3}}$ which decreased with Cr-doping. The optical conductivity increased with photon energy near the absorption band edge. This was due to the optimal absorption via electron excitation of the photon energy^59^ which reduced the free carriers. The optical conductivity was reduced in the lower energy region (visible absorption), which showed the localization of the initially free carriers. The optical conductivity increased with Cr-doping in $\text{MgO}{-}{\text{Bi}}_{2}{\text{O}}_{3}$ lattice.

#### Real and imaginary parts of dielectric constant

The real part of the dielectric constant, ${\varepsilon }_{r}={n}^{2}-{k}^{2}$ indicates the ability of a material to reduce the speed of light. The imaginary part, ${\varepsilon }_{i}=2nk$ gives the absorption of energy due to dipole motion from an electric field $\left[54\right].$ The ratio $\frac{{\varepsilon }_{i}}{{\varepsilon }_{r}} =\text{ tan}\delta$ gives information about loss factor. Figure 9b–d show the real and imaginary parts of the dielectric constant and loss factor on photon energy. The real part are higher than the imaginary part of the dielectric constant. *ε*_r_ of the dielectric constant indicates the real electrical energy saving and *ε*_i_ shows the absorption loss related to free carriers. The curves of the two parts are naturally oscillatory and relies on the crystal structure^60^. The real part rises and falls and this is similar to refractive index where the effect of *k* can be omitted. The profile of the imaginary part was similar to the extinction coefficient. The loss factor was nearly constant in the energy range 2.2 to 2.9 eV but increase pronouncedly near 2.9 eV.

#### Optical band gap (*E*_*g*_)

The absorption spectrum can determine the optical band gap, *E*_*g*_ by using the Tauc plot method^42^:

15 $${({\upalpha {\text{h}}\upupsilon })}^{\text{n}}=A \left(\text{h}\upupsilon-{E}_{g}\right),$$

where, *ʋ* is incident frequency, *A* is a constant that depends on electron and hole effective masses. The exponent *n* = 1/2, 2, 3/2 or 3 for direct, indirect, forbidden direct, and forbidden indirect transitions, respectively^61^.

**Figure 10:**
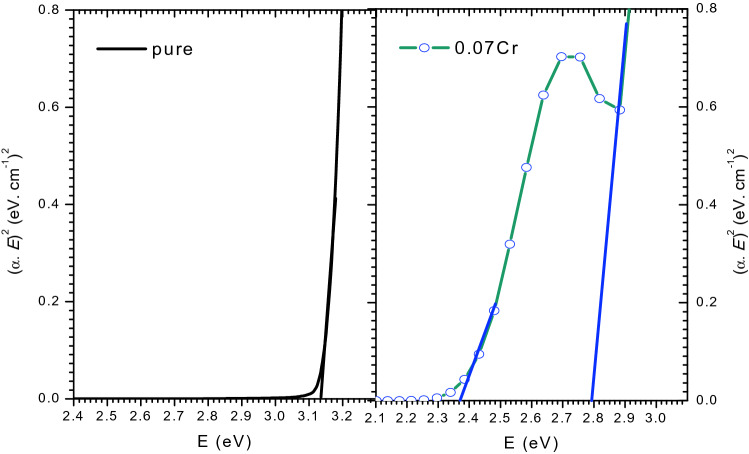
Optical direct transition band gap energy (*E*_g_) for MgO–Bi_2−*x*_Cr_*x*_O_3_ nanocomposite for *x* = 0 and 0.07.

The plot of (${\alpha h\nu }^{2}$) versus $h\nu$ can be used to obtain ${E}_{g}$ of the samples (Fig. 10). The plot exhibits two different slopes. ${E}_{g}$ values for $\text{MgO}{-}{\text{Bi}}_{2-\text{x}}{Cr}_{x}{\text{O}}_{3}$ nanocomposite are listed in Table 3. The Cr-doped $\text{MgO}{-}{\text{Bi}}_{2}{\text{O}}_{3}$ nanocomposites exhibited two energy gaps at 2.36 and 2.76 eV. The second energy gap may be due to ${\text{BiCrO}}_{3}$. The optical band gap decreased when Cr was doped (red shift), suggesting that Cr^3+^ in the valence band acted as defects which reduced the band gap. The smaller gap with Cr-doping was due to the impurities and increase in free electrons. The excitations occurred from the filled valence band to impurity energy levels^62^.

**Table 3:** Direct transition band gap *E*_g_ values of MgO–Bi_2−*x*_Cr_*x*_O_3_ nanocomposite for *x* = 0 and 0.07.

	Band gap, ${E}_{g}$ (eV)	Band gap, ${E}_{g}$ (eV)
*x* = 0	3.14	–
*x* = 0.07	2.77	2.37

Cr^3+^ replacing Bi^3+^ sites will create more defects or impurities in the electronic energy gap of $\text{MgO}{-}{\text{Bi}}_{2}{\text{O}}_{3}$ structures. It may be reason for the decrease in the energy gap and the appearance of the second energy gap. This energy gap was smaller than the undoped $\text{MgO}{-}{\text{Bi}}_{2}{\text{O}}_{3}$ (3.14 eV) (Fig. 10). The decrease in energy gap value indicated an increase in photocatalytic efficiency. Previous studies have also reported two energy band gap^63–65^.

### Antibacterial activity

**Figure 11:**
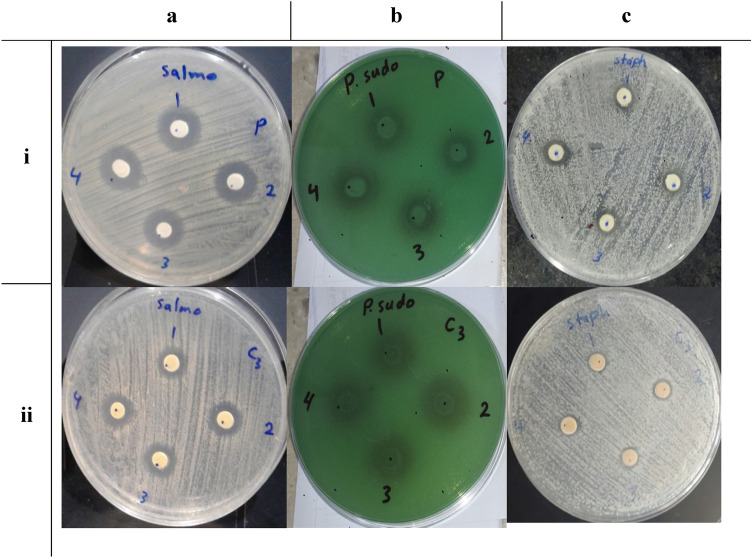
Bacterial growth inhibition activity of MgO–Bi_2−*x*_Cr_*x*_O_3_ nanocomposites at different doping of Cr. Rows refer to (i) *x* = 0 and (ii) *x* = 0.07. Columns refer to bacteria (**a**) *S. typhosa*, (**b**) *P. aeriginosa* and (**c**) *S. aureus*.

In the present investigation, $\text{MgO}{-}{\text{Bi}}_{2-x}{\text{Cr}}_{x}{\text{O}}_{3}$ nanocomposites for *x* = 0 and 0.07 with 50, 25, 12.5, 6.25 mg/ml dilutions were prepared and the antibacterial activity was determined against gram-negative bacteria (*Salmonella typhimurium, Pseudomonas aeruginosa*) and gram-positive bacteria (*Staphylococcus aureus*) (Table 4). The images of antibacterial studies of $\text{MgO}{-}{\text{Bi}}_{2-x}{\text{Cr}}_{x}{\text{O}}_{3}$ nanocompositesagainst *S. typhimurium, P. aeriginosa and S. aureus* bacterial pathogens are shown in Fig. 11.

**Table 4:** Antibacterial activity of MgO–Bi_2−*x*_Cr_*x*_O_3_ nanocomposite for *x* = 0 and 0.07 against gram positive and gram negative bacteria.

Microorganisms	Zone of inhibition (mm) at concentration in (µg/ml)		
	Con	*x* = 0	*x* = 0.07
*Salmonella typhimurium* (Gram negative)	S1	15	10
	S2	15	11
	S3	15	11
	S4	15	11
*Pseudomonas aeruginosa* (Gram negative)	S1	14	14
	S2	13	15
	S3	14	12
	S4	15	12
*Staphylococcus aureus* (Gram positive)	S1	9	7
	S2	8	9
	S3	8	8
	S4	11	7

The antibacterial efficiency was determined by measuring the diameter of the inhibition zone around the disc using antibiotic zone scale in millimeter. Cr-doped $\text{MgO}{-}{\text{Bi}}_{2}{\text{O}}_{3}$ showed lower antimicrobial activity than non-doped $\text{MgO}{-}{\text{Bi}}_{2}{\text{O}}_{3}$. The inhibition zone has more bacteria *S. typhimurium*, *P. aeriginosa* than *S. aureus*. The active species from Cr-doping and reduced crystallite size has direct effect on the cellular inhibition. Surface morphology also gave a higher antibacterial activity. The enhanced antibacterial performance of the nano size particle was attributed to the high surface charge and reactive oxygen species (ROS) formation^66^. A strong antibacterial activity against gram-negative was also due to the increase in H_2_O_2_ produced from $\text{MgO}{-}{\text{Bi}}_{2}{\text{O}}_{3}$ surface which is lethal to the bacteria^67^. On the other hand, this might also be due to the difference in the cell structure of the bacteria. As the gram-positive bacteria have a thick lipopolysaccharide cell membrane as related to gram-negative bacteria^68–72^. The production of heavy metallic ions (${\text{Bi}}^{3+}$, ${\text{Mg}}^{2+}$ and ${\text{Cr}}^{3+}$) is the second reason for the antibacterial activity. Because of the presence of thiol group (–SH) in the proteins on outer surface of the cell membrane, the metallic ions ${\text{Bi}}^{3+}$, ${\text{Mg}}^{2+}$ and ${\text{Cr}}^{3+}$ are attracted to the cell membrane. The metallic ions then penetrate the cell membrane and denature proteins, hence, causing damage to the membrane of the bacterial cell. In addition, the texture of the nanocomposite surface causes mechanical damage to the membrane^72–76^.

In this regard, surface defects such as oxygen vacancies and Bi, Mg and Cr interstitial defects have been reported to be the most crucial and matter of intensive research^72^. As in our case, the shape remains unchanged with addition of Cr content and the only variation is in the defects densities as suggested by SEM results.

## Conclusions

Samples with nominal starting compositions MgO–Bi_2−*x*_Cr_*x*_O_3_ (*x* = 0 and 0.07) nanocomposites were prepared by using a low-cost solvent-deficient method. The phase of MgO–Bi_2−*x*_Cr_*x*_O_3_ was estimated using XRD method which showed that the samples were dominantly monoclinic crystalline structure of $\alpha{\text{-}}{\text{Bi}}_{2}{\text{O}}_{3}$ phase at *x* = 0 and there was no peaks attributed to MgO in the composite. Partial substitutions of Cr in place of Bi in $\text{MgO}{-}{\text{Bi}}_{2}{\text{O}}_{3}$ showed the tetragonal ${\text{BiCrO}}_{3}$ phase. Partial substitution of Cr in place of Bi in $\text{MgO}{-}{\text{Bi}}_{2}{\text{O}}_{3}$ showed a decrease in the crystallite size of $\text{MgO}{-}{\text{Bi}}_{2}{\text{O}}_{3}$. SEM micrographs showed that the grains were aggregated and uniformly distributed with micrometer size. EDXA revealed the elemental composition of MgO–Bi_2−*x*_Cr_*x*_O_3_. Uv–Vis spectra showed that MgO–Bi_2−*x*_Cr_*x*_O_3_ can be applied in optoelectronics, photonic and optical communication. Direct transition ${E}_{g}$ decreased with Cr-doping in place of Bi in $\text{MgO}{-}{\text{Bi}}_{2}{\text{O}}_{3}$ from 3.14 to 2.77 eV. The Cr-doped $\text{MgO}{-}{\text{Bi}}_{2}{\text{O}}_{3}$ exhibited two energy gaps at 2.36 and 2.76 eV. Cr-doping decreased the inhibitory activity of MgO–Bi_2−*x*_Cr_*x*_O_3_ nanocomposite against the different types of bacteria.

## Data Availability

The datasets generated and/or analysed during the current study are available from the corresponding author on reasonable request.

## References

[1] Wahab R, Ansari SG, Dar MA, Kim YS, Shin HS (2007). Synthesis of magnesium oxide nanoparticles by sol-gel process. Mater. Sci. Forum.

[2] Ren Ao, Liu C, Hong Y, Shi W, Lin S, Li P (2014). Enhanced visible-light-driven photocatalytic activity for antibiotic degradation using magnetic NiFe_2_O_4_/Bi_2_O_3_ heterostructures. Chem. Eng. J..

[3] Shannon RD (1976). Revised effective ionic radii and systematic studies of interatomic distancesinhalidesandchalcogenides. Acta Cryst. Sect. A.

[4] Sood S, Umar A, Mehta SK, Kansal SK (2015). α-Bi_2_O_3_ nanorods: An efficient sunlight active photocatalyst for degradation of Rhodamine B and 2, 4, 6-trichlorophenol. Ceram. Int..

[5] Yan Y, Zhou Z, Cheng Y, Qiu L, Gao C, Zhou J (2014). Template-free fabrication of α-and β-Bi_2_O_3_ hollow spheres and their visible light photocatalytic activity for water purification. J. Alloy. Compds..

[6] Maruthamuthu P, Gurunathan K, Subramanian E, Sastri M (1994). Visible light-induced hydrogen production from water with Pt/Bi_2_O_3_/RuO_2_ in presence of electron relay and photosensitizer. Int. J. Hydrogen Energy.

[7] Gurunathan K (2004). Photocatalytic hydrogen production using transition metal ions-doped γ-Bi_2_O_3_ semiconductor particles. Int. J. Hydrogen Energy.

[8] Bian Z, Zhu J, Wang S, Cao Y, Qian X, Li H (2008). Self-assembly of active Bi_2_O_3_/TiO_2_ visible photocatalyst with ordered mesoporous structure and highly crystallized anatase. J. Phys. Chem. C.

[9] Fan H, Teng X, Pan S, Ye C, Li G, Zhang L (2005). Optical properties of δ-Bi_2_O_3_ thin films grown by reactive sputtering. Appl. Phys. Lett..

[10] Leontie L, Caraman M, Alexe M, Harnagea C (2002). Structural and optical characteristics of bismuth oxide thin films. Surf. Sci..

[11] Leontie L, Caraman M, Rusu G (2000). On the photoconductivity of Bi_2_O_3_ in thin films. J. Optoelectr. Adv. Mater..

[12] López-Salinas F, Martínez-Castañón G, Martínez-Mendoza J, Ruiz F (2010). Synthesis and characterization of nanostructured powders of Bi_2_O_3_, BiOCl and Bi. Mater. Lett..

[13] Takeyama T, Takahashi N, Nakamura T, Ito S (2004). Growth of the high reflectivity Bi_2_O_3_ glass films by atmospheric pressure halide CVD. Opt. Mater..

[14] Thayer R, Randall CA, Trolier-Mckinstry S (2003). Medium permittivity bismuth zinc niobate thin film capacitors. J. Appl. Phys..

[15] Adamian Z, Abovian H, Aroutiounian V (1996). Smoke sensor on the base of Bi_2_O_3_ sesquioxide. Sens. Actuators B Chemical.

[16] Aspiala M, Sukhomlinov D, Taskinen P (2014). Standard thermodynamic properties of Bi_2_O_3_ by a solid-oxide electrolyte EMF technique. J. Chem. Therm..

[17] Bhande SS, Mane RS, Ghule AV, Han S-H (2011). A bismuth oxide nanoplate-based carbon dioxide gas sensor. Script. Mater..

[18] Ghedia S, Locherer T, Dinnebier R, Prasad D, Wedig U, Jansen M, Senyshyn A (2010). High-pressure and high-temperature multianvil synthesis of metastable polymorphs of Bi_2_O_3_: Crystal structure and electronic properties. Phys. Rev. B.

[19] Gujar T, Shinde V, Lokhande C, Han S-H (2006). Electrosynthesis of Bi_2_O_3_ thin films and their use in electrochemical supercapacitors. J. Power Sources.

[20] Hanna TA (2004). The role of bismuth in the SOHIO process. Coord. Chem. Rev..

[21] Oprea I-I, Hesse H, Betzler K (2004). Optical properties of bismuth borate glasses. Opt. Mat..

[22] Wachsman ED, Lee KT (2011). Lowering the temperature of solid oxide fuel cells. Science.

[23] Li E-J, Xia K, Yin S-F, Dai W-L, Luo S-L, Chak-Tong Au (2011). Preparation, characterization and photocatalytic activity of Bi_2_O_3_–MgO composites. Mater. Chem. Phys..

[24] Li LZ, Yan B (2009). CeO_2_-Bi_2_O_3_ nanocomposite: Two step synthesis, microstructure and photocatalytic activity. J. Non-Cryst. Solids.

[25] Jan T, Azmat S, Wahid B, Adil M, Alawadhi H, Mansoor Q, Farooq Z, Ilyas SZ, Ahmad I, Ismail M (2018). Chemically synthesized ZnO-Bi_2_O_3_(BZO) nanocomposites with tunable optical, photoluminescence and antibacterial characteristics. Mater. Sci. Semicond. Proc..

[26] Wu Z, Guo G, Xu M, Shi Y (2014). Low-temperature synthesis of ZnO-Bi_2_O_3_ nanocomposite by sonochemical route. Int. J. Nanomanuf..

[27] Hernandez-Delgadillo R, Velasco-Arias D, Martinez-Sanmiguel JJ, Diaz D, Zumeta-Dube I, Arevalo-Niño K, Cabral-Romero C (2013). Bismuth oxide aqueous colloidal nanoparticles inhibit Candida albicans growth and biofilm formation. Int. J. Nanomed..

[28] Qin F, Zhao H, Li G, Yang H, Li J, Wang R, Liu Y, Hu J, Sun H, Chen R (2014). Size-tunable fabrication of multifunctional Bi_2_O_3_ porous nanospheres for photocatalysis, bacteria inactivation and template-synthesis. Nanoscale.

[29] Riente P, Matas Adams A, Albero J, Palomares E, Pericas MA (2014). Light-driven organocatalysis using inexpensive, nontoxic Bi2O3 as the photocatalyst. Angew. Chem..

[30] Spoto G, Gribov E, Ricchiardi G, Damin A, Scarano D, Bordiga S, Lamberti C, Zecchina A (2004). Carbon monoxide MgO from dispersed solids to single crystals: A review and new advances. Prog. Surf. Sci..

[31] Varshney D, Dwivedi S (2015). On the synthesis, structural, optical and magnetic properties of nano-size Zn–MgO. Superlattices Microstruct..

[32] Richards R, Li W, Decker S, Davidson C, Koper O, Zaikovski V, Volodin A, Rieker T, Klabunde KJ (2000). Consolidation of metal oxide nanocrystals. Reactive pellets with controllable pore structure that represent a new family of porous, inorganic materials. J. Amer. Chem. Soc..

[33] Sawai J, Kojima H, Igarashi H, Hashimoto A, Shoji S, Sawaki T, Hakoda A, Kawada E, Kokugan T, Shimizu M (2000). Antibacterial characteristics of magnesium oxide powder. World J. Microbiol. Biotechnol..

[34] Krishnamoorthy K, Moon JY, Hyun HB, Cho SK, Kim S-J (2012). Mechanistic investigation on the toxicity of MgO nanoparticles toward cancer cells. J. Mater. Chem..

[35] Zhang W, Tay HL, Lim SS, Wang Y, Zhong Z, Xu R (2010). Supported cobalt oxide on MgO: highly efficient catalysts for degradation of organic dyes in dilute solutions. Appl. Catal. B Environ..

[36] Tang Z-X, Fang X-J, Zhang Z-L, Zhou T, Zhang X-Y, Shi L-E (2012). Nanosize MgO as antibacterial agent: preparation and characteristics. Braz. J. Chem. Eng..

[37] Kandjani AE, Tabriz MF, Moradi OM, Mehr HR, Kandjani SA, Vaezi M (2011). An investigation on linear optical properties of dilute Cr doped ZnO thin films synthesized via sol–gel process. J. Alloy. Compds..

[38] Naray-Szabo I (1947). The perovskite-structure family. Muegyetemi Kozlemenyek.

[39] Kaur P, Kumar S, Negi N, Rao S (2015). Enhanced magnetism in Cr-doped ZnO nanoparticles with nitrogen co-doping synthesized using sol–gel technique. Appl. Nanosci..

[40] Popescu T, Lupu A, Feder M, Tarabasanu-Mihaila D, Teodorescu V, Vlaicu A, Diamandescu L (2014). In vitro toxicity evaluation of Ti^4+^-stabilized γ-Bi_2_O_3_ sillenites. Toxicol. In Vitro.

[41] Jeejamol DJ, Raj AME, Jayakumari K, Ravidhas C (2018). Optimization of CdO nanoparticles by Zr^4+^ doping for better photocatalytic activity. J. Mater. Sci. Mater. Electron..

[42] AL-Osta A, Alnehia A, Qaid AA, Al-Ahsab HT, Al-Sharabi A (2020). Structural, morphological and optical properties of Cr doped ZnS nanoparticles prepared without any capping agent. Optik-Int. J. Light Electr. Opt..

[43] Sahay P, Nath R (2008). Al-doped ZnO thin films as methanol sensors. Sens. Actuators B Chem..

[44] Bhargava R, Sharma PK, Kumar S, Pandey AC, Kumar N (2010). Consequence of doping mediated strain and the activation energy on the structural and optical properties of ZnO: Cr nanoparticles. J. Solid Stat. Chem..

[45] Maensiri S, Laokul P, Promarak V (2006). Synthesis and optical properties of nanocrystalline ZnO powders by a simple method using zinc acetate dihydrate and poly (vinyl pyrrolidone). J. Cryst. Growth.

[46] Hassan MM, Khan W, Azam A, Naqvi A (2015). Influence of Cr incorporation on structural, dielectric and optical properties of ZnO nanoparticles. J. Ind. Eng. Chem..

[47] Kumar S, Mukherjee S, Kr Singh R, Chatterjee S, Ghosh A (2011). Structural and optical properties of sol-gel derived nanocrystalline Fe-doped ZnO. J. Appl. Phys..

[48] Smithard M (1973). Size effect on the optical and paramagnetic absorption of silver particles in a glass matrix. Solid State Commun..

[49] Tarwal N, Shinde V, Kamble A, Jadhav P, Patil D, Patil V, Patil P (2011). Photoluminescence and photoelectrochemical properties of nanocrystalline ZnO thin films synthesized by spray pyrolysis technique. Appl. Surf. Sci..

[50] Al-Dahash GAW, Najeeb HN, Baqer A, Tiama R (2011). The Effect of Bismuth Oxide Bi2O3 on Some Optical Properties of Poly-vinyl Alcohol.

[51] Hafiz M, El-Kabany N, Kotb HM, Bakier Y (2015). Determination of optical band gap and optical constants of Ge_*x*_Sb_40−__*x*_Se_60_ thin films. Int. J. Thin Films Sci. Technol..

[52] Al-Sharabi A, Alnehia A, Al-Osta A, Nabil Yahya AA (2019). Effect of copper doping on structural and optical properties of zinc sulfide (ZnS) nanoparticles. Al-Baydha Univ. J. Res..

[53] Hosni H, Fayek S, El-Sayed S, Roushdy M, Soliman M (2006). Optical properties and DC electrical conductivity of Ge_28−__*x*_Se_72_Sb_*x*_ thin films. Vacuum.

[54] Abdulwahab AM (2021). Asma’a Ahmed AL-Adhreai & Abdullah Ahmed Ali Ahmed, Influence of Ni-Co dual doping on structural and optical properties of CdSe thin films prepared by chemical bath deposition method. Optik Int. J. Light Electr. Opt..

[55] Ávan der Put PJ (1996). Morphology control of thin LiCoO_2_ films fabricated using the electrostatic spray deposition (ESD) technique. J. Mater. Chem..

[56] Barman P (2010). An optical study of vacuum evaporated Se_85−__*x*_Te_15_Bi_*x*_ chalcogenide thin films. Physica B.

[57] Madhup D, Subedi D, Chimouriy S (2010). Optical characterization and thickness estimation of Al^3+^ ion doped ZnO nanofilms from transmittance spectra. J Optoelectr. Adv. Mater..

[58] Millis A, Zimmers A, Lobo R, Bontemps N, Homes C (2005). Mott physics and the optical conductivity of electron-doped cuprates. Phys. Rev. B.

[59] Habubi N, Oboudi S, Chiad S (2012). Study of some optical properties of mixed SnO_2_-CuO thin films. J. Nano Electr. Phys..

[60] Khalaf MK, Al-Kader DSA, Salh JM (2021). Effect of thickness and type of substrate on optical properties of chromium oxide thin film prepared by sputtering magnetron. IOP Conf. Ser. Mater. Sci. Eng..

[61] Al-Sharabi A, Al-Hussam AM, Abdullh SKS (2019). Synthesis and characterization of metal complexes of Cu (ii) and Cd (ii) with poly vinyl alcohol and studied of electrical and optical properties. Int. J. Multidisc. Res. Dev..

[62] Ghomrani F, Aissat A, Arbouz H, Benkouider A (2015). Al concentration effect on ZnO based thin films: For photovoltaic applications. Energy Procedia.

[63] Ramola R, Rawat M, Joshi K, Das A, Gautam SK, Singh F (2017). Study of phase transformation induced by electronic excitation in pure and yttrium doped ZrO_2_ thin films. Mater. Res. Exp..

[64] Merupo V-I, Velumani S, Ordon K, Errien N, Szade J, Kassiba A-H (2015). Structural and optical characterization of ball-milled copper-doped bismuth vanadium oxide (BiVO_4_). CrystEngComm.

[65] Ho C-T, Weng T-H, Wang C-Y, Yen S-J, Yew T-R (2014). Tunable band gaps of Co_3−__*x*_Cu_*x*_O_4_ nanorods with various Cu doping concentrations. RSC Adv..

[66] Ma J, Liu C, Yan K (2022). CQDs-MoS_2_ QDs loaded on Dendritic fibrous nanosilica/hydrophobic waterborne polyurethane acrylate for antibacterial coatings. Chem. Eng. J..

[67] Liu Y, Ki H-I (2012). Characterization and antibacterial properties of genipin crosslinked chitosan/poly (ethylene glycol)/ZnO/Ag nanocomposites. Carbohyd. Polym..

[68] El-Batal AI, Al-Hazmi NE, Mosallam FM, El-Sayyad GS (2018). Biogenic synthesis of copper nanoparticles by natural polysaccharides and *Pleurotus ostreatus* fermented fenugreek using gamma rays with antioxidant and antimicrobial potential towards some wound pathogens. Microb. Pathog..

[69] He Y, Ingudam S, Reed S, Gehring A, Strobaugh TP, Irwin P (2016). Study on the mechanism of antibacterial action of magnesium oxide nanoparticles against foodborne pathogens. J. Nanobiotechnol..

[70] Verma SK, Jha E, Panda PK, Das JK, Thirumurugan A, Suar M, Parasha SKS (2018). Molecular aspect of core-shell intrinsic defect induced enhanced antibacterial activity of ZnO nanocrystal. Nanomedicine.

[71] Franklin NM, Rogers NJ, Apte SC, Batley GE, Gadd GD, Casey PS (2007). Comparative toxicity of nanoparticulate ZnO, bulk ZnO, and ZnCl_2_ to a freshwater microalga (*Pseudokirchneriella subcapitatata*): The importance of particle solubility. Environ. Sci. Technol..

[72] Akhavan O, Mehrabian M, Mirabbaszades K, Azimirad R (2009). Hydrothermal synthesis of ZnO nanorod array for photocatalytic inactivation of bacteria. J. Phys. D Appl. Phys..

[73] Schneider JJ, Hoffmann RC, Engstler J, Klyszcz A, Erdem E, Jakes P, Eichel RA, Bauermann LP, Bill J (2010). Synthesis, characterization, defect chemistry, and FET properties of microwave-derived nanoscaled zinc oxide. Chem. Mater..

[74] Xia T, Kovochich M, Liong M, Mädler L, Gilbert B, Shi H, Yeh JI, Zink JI, Nel AE (2008). Comparison of the mechanism of toxicity of zinc oxide and cerium oxide nanoparticles based on dissolution and oxidative stress properties. ACS Nano.

[75] Karthik K, Dhanuskodi S, Gobinath C, Prabukumar S, Sivaramakrishnan S (2018). Multifunctional properties of microwave assisted CdO–NiO–ZnO mixed metal oxide nanocomposite: Enhanced photocatalytic and antibacterial activities. J. Mater. Sci. Mater. Electron..

[76] Tang J, Chen Q, Ligenr Xu, Zhang S, Feng L, Cheng L, Huan Xu, Liu Z, Peng R (2013). Graphene oxide-silver nanocomposite as a highly effective antibacerial agent with species-specific mechanisms. ACS Appl. Mater. Interfaces.

